# Fast acquisition of propagating waves in humans with low-field MRI: Toward accessible MR elastography

**DOI:** 10.1126/sciadv.abo5739

**Published:** 2022-09-09

**Authors:** Maksym Yushchenko, Mathieu Sarracanie, Najat Salameh

**Affiliations:** Center for Adaptable MRI Technology (AMT Center), Department of Biomedical Engineering, University of Basel, Allschwil, Switzerland.

## Abstract

Most commonly used at clinical magnetic fields (1.5 to 3 T), magnetic resonance elastography (MRE) captures mechanical wave propagation to reconstruct the mechanical properties of soft tissue with MRI. However, in terms of noninvasively assessing disease progression in a broad range of organs (e.g., liver, breast, skeletal muscle, and brain), its accessibility is limited and its robustness is challenged when magnetic susceptibility differences are encountered. Low-field MRE offers an opportunity to overcome these issues, and yet it has never been demonstrated in vivo in humans with magnetic fields <1.5 T mainly because of the long acquisition times required to achieve a sufficient signal-to-noise ratio. Here, we describe a method to accelerate 3D motion-sensitized MR scans at 0.1 T using only 10% *k*-space sampling combined with a high-performance detector and an efficient encoding acquisition strategy. Its application is demonstrated in vivo in the human forearm for a single motion-encoding direction in less than 1 min.

## Teaser

Tailored, fast acquisition schemes are used to perform in vivo low-field MR elastography scans in humans.

## INTRODUCTION

Magnetic resonance elastography (MRE) is an MR-based technique for the noninvasive in vivo quantification of mechanical tissue properties (*1*, *2*). Typically, MRE techniques involve the generation of a mechanical vibration associated with motion-sensitized MR sequences and ad hoc reconstruction algorithms, generally confined to high–magnetic field regimes in preclinical and clinical settings. Research and commercial low-field MRI scanners (≤~0.2 T) have recently regained attention as a complement to conventional clinical scanners (1.5 to 3 T), the former of which offer various advantages, including simpler installation, less maintenance, and potential for smaller physical footprints (*3*). MRI can thus become more accessible and less expensive and provide a more efficient workflow in health care management, leveraging both whole-body MR systems and versatile and mobile-dedicated devices in point-of-care settings. From a physics point of view, low-field MRI suffers less, if not at all, from susceptibility artifacts. This is particularly relevant for MRE since its scans typically use longer echo times (TEs) than standard imaging sequences to accommodate the duration of motion-encoding gradients (MEGs). As a consequence, at higher clinical fields, this technique is notably sensitive to shortened T2* (*4*) because of iron overload that typically occurs in chronic liver diseases (CLDs) (*5*–*9*), near implants, or at air-tissue interfaces near the sinuses or in the lung. In addition, MRE can benefit from other inherent low-field advantages such as lower specific absorption rates, reduced chemical shift, maximized T2* thanks to more homogeneous static magnetic fields for a given scanner geometry, shorter T1, and improved MR compatibility helping locate mechanical excitation devices not far from the scanner. Several applications could directly leverage such aspects, including patients with CLDs and liver iron overload with whole-body low-field MR scanners or musculoskeletal (MSK) MRE in compact, dedicated extremities or weight-bearing scanners. However, standard (i.e., high-field) MRE approaches may require signal averaging to achieve a sufficient signal-to-noise ratio (SNR) in a low-field scenario, leading to longer acquisition times. This is one of the main reasons why low-field MRE has never been performed in vivo in humans, although some studies can be found in phantoms and tissue samples with tabletop medium-field 0.5-T systems (*10*–*12*), in phantoms at ultralow-field 6.5 mT (*13*) or in phantoms and animals (ex vivo and in vivo) at 0.1 T (*14*–*16*).

Low-field MRI does provide intrinsically lower net magnetization when compared to higher fields. However, the resulting SNR penalty can be mitigated by focusing on different aspects of the MR workflow, such as electromagnetic noise (*3*, *17*, *18*). For instance, at low frequencies, the noise regime is often coil-dominated, and optimizing the radio frequency (RF) detector from this perspective can bring greater benefits than in sample-dominated noise regimes (*19*–*21*). While two-dimensional (2D) acquisitions provide little SNR at low field, 3D sequences can be used more efficiently thanks to shorter T1 recovery in tissue, which allows for short repetition times (TRs) of tens of milliseconds with low–flip angle gradient echo sequences. At low field, the SNR of 3D acquisitions greatly benefits from the signal being received from a large volume rather than from thinner slices. In 3D, further acceleration and noise reduction can be achieved with undersampling patterns along the two phase-encoding (PE) directions in *k*-space (*13*, *22*). 3D acquisitions can present an additional advantage for MRE reconstruction techniques, allowing a continuous volume to be probed with isotropic resolution (*23*) and thereby avoiding the potential issue of 2D multislice profile accuracy and contiguity. However, true 3D sequences (i.e., non-multislice or multislab) have been used for MRE in very few instances (*13*) and then mostly with non-Cartesian acceleration approaches in vivo (*24*, *25*). The main reason for this paucity is that standard Cartesian 3D acquisitions with MRE are considered time-consuming in comparison to other methods because of the relatively long TR (100 to 200 ms) used at high fields (*24*, *26*).

The wavelength of human MRE shear waves in vivo is typically of the order of centimeters in the common range of vibration frequencies (30 to 150 Hz) and shear stiffness of most organs (few kilopascals). Such waves are usually acquired with several voxels per wavelength, and they are thus encoded by relatively low spatial frequency information in the acquired *k*-space, as highlighted in previous works (*27*, *28*). In such a context, it is fair to challenge the total amount of information relevant to acquire, while a strategy that focuses on sampling only a limited *k*-space region could have a twofold benefit. First, the sequence could be drastically accelerated, especially in the case of 3D acquisitions that are not only particularly advantageous at low field but also whereby both PE directions could be conveniently undersampled to achieve high acceleration factors. Second, noise, the impact of which is more prominent at higher spatial frequencies, would be inherently reduced. In this way, instead of postprocessing filters commonly applied in MRE reconstruction pipelines, *k*-space undersampling would provide an intrinsic filter during acquisition while benefitting from drastically reduced scan time.

In this work, we show that it is possible to retrieve the shear wave propagation for stiffness estimation at 0.1 T by using an optimized double-channel RF coil, along with a smart acquisition scheme that samples only 10% of a Cartesian 3D *k*-space. This approach is validated in a silicone phantom and applied to human extremity muscles in vivo, with a short scan time of less than 1 min per dataset, sufficient for manual wavelength estimation, and an additional 30 s per additional time point. Our undersampling strategy thus opens the way for accelerated 3D motion-encoding acquisitions and fast in vivo MR elastography, which could be leveraged regardless of magnetic field strength.

## RESULTS

### Phantom experiments

**Fig. 1.**
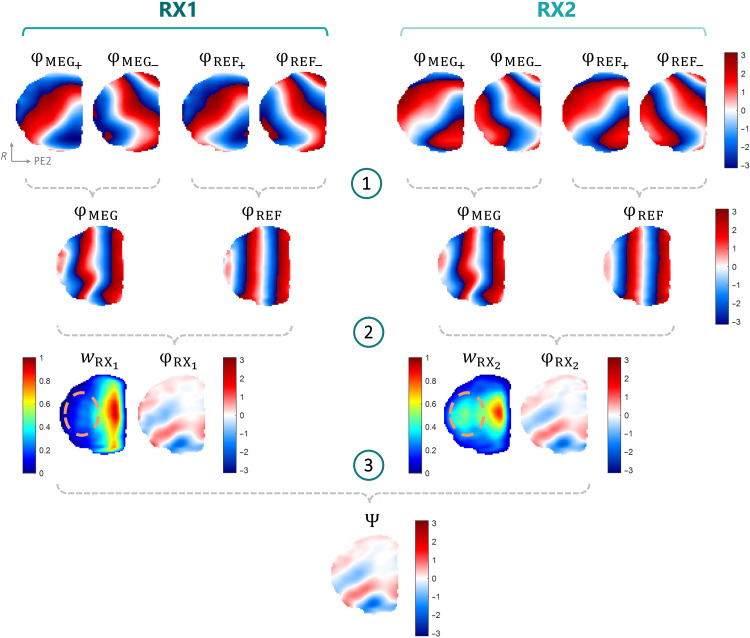
Data processing pipeline. Data processing steps from motion-encoded complex data ξ to final wave data Ψ, with the example of the sagittal slice 55/79 (R-PE2 plane) for a 10%-sampled phantom acquisition. R, readout direction; PE2, phase-encoding direction 2. All color scales for the phase maps range from −π to +π. For each channel, subtracting datasets with opposite MEG polarities (step 1) provides double motion-encoding sensitivity and removes the contribution of possible *B*_0_ and eddy current phase variations. A spurious phase gradient along the MEG direction (PE2) is visible in φ_REF_, which should otherwise be flat when no motion is present. In step 2, φ_REF_ is then subtracted from φ_MEG_, which helps obtain wave information only. This wave information from the two channels is then combined (step 3) via a weighted summation using the respective normalized coil sensitivities *w*_RX_1__ and *w*_RX_2__ to obtain final wave data Ψ.

Figure 1 shows an example of the processing pipeline allowing the extraction of motion-encoded phase information (i.e., steps 1 to 3) in a phantom. The notations and symbols in Fig. 1, mentioned in this paragraph, are further described in the “Data processing” section. We observe that the reference phase φ_REF_ (calculated in step 1) is not flat, and it is perturbed by a gradient along the MEG direction, similar to φ_MEG_. As illustrated in step 2, subtracting the reference phase helps correct this spurious phase gradient, which no longer appears on φ_RX_. Last, the two channels are combined (step 3). It is notable that the final phase image Ψ benefits from higher accuracy because of the combination of the two receive channels that exhibit different, and complementary, sensitivity profiles (step 3, this effect is notable in the regions of low RX1 channel sensitivity *w*_RX1_, nicely complemented by higher sensitivity from *w*_RX2_ and highlighted with an added dotted-line region of interest).

**Fig. 2.**
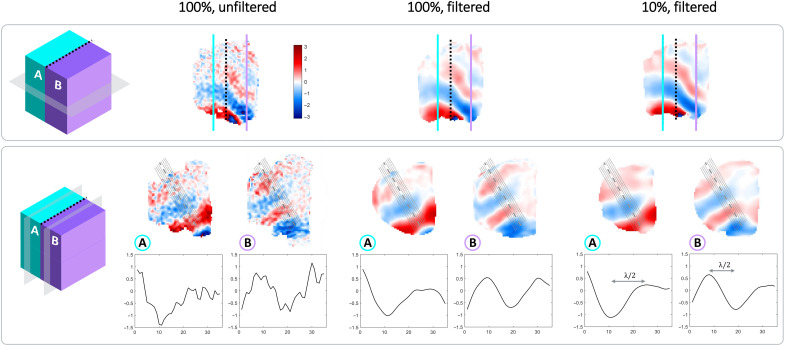
Phantom wave results. Comparison of datasets acquired in the phantom, first time point at 49-Hz vibration: 100%-sampled and unfiltered wave data (100%, unfiltered), 100%-sampled and filtered wave data (100%, filtered), and 10%-sampled and filtered wave data (10%, filtered). First row: Coronal slice 31/59, with a black dotted line indicating the separation between the phantom compartments; solid colored lines indicate the location of sagittal slices (row 2). Second row: Sagittal slices in each phantom compartment (30/79 in the stiffer compartment A and 56/79 in the softer compartment B). The dotted lines on the sagittal view indicate the segments considered for wavelength estimation from the wave profiles. Below each sagittal slice, wave profiles along the middle segment (dashed line, sagittal views) in the corresponding compartments are represented. Wavelength and shear stiffness estimations are reported in Table 2, while wave amplitudes for each profile are reported in Table 3.

The comparison between a 100%-sampled dataset, i.e., both unfiltered and filtered with a 3D Tukey window (*r* = 1, as described in the Data processing section), and a 10%-sampled dataset in the phantom is illustrated in Fig. 2. Overall, the three cases produce similar wave patterns (cf. wave profiles in Fig. 2). The coronal view shows the different wave patterns in the two compartments with different stiffnesses. The wavelength and shear stiffness obtained from the wave profiles extracted from sagittal views are presented in Table 1, while Table 2 presents the wave amplitude corresponding to each profile. Table 3 summarizes the SNR over the phantom for the analyzed datasets. SNR is overall higher in the 100%-filtered and 10%-filtered datasets than in the 100%-unfiltered one. For each acquisition and channel, the voxel-wise SNR was observed to be always within two SDs of the mean of the reference datasets.

**Table 1.** Phantom results. Wavelength and shear stiffness estimated as mean and SD across all profiles in phantom compartments A and B (Fig. 2) at 49-Hz vibration.

**Dataset**	**Wavelength**	**Shear stiffness**
100% unfiltered comp. A | comp. B	64.4 ± 20.2 | 39.5 ± 7.2 mm	10.8 ± 6.2 | 3.9 ± 1.5 kPa
100% filtered comp. A | comp. B	64.0 ± 3.9 | 37.8 ± 3.8 mm	9.9 ± 1.2 | 3.5 ± 0.7 kPa
10% filtered comp. A | comp. B	57.1 ± 4.2 | 43.4 ± 2.6 mm	7.9 ± 1.2 | 4.5 ± 0.5 kPa

**Table 2.** Phantom wave profiles. Wave amplitude between a positive peak and a negative peak for each extracted wave profile in phantom compartments A and B (Fig. 2) at 49-Hz vibration.

**Dataset**		**Wave amplitude (rad)**								
		**Profile 1**	**Profile 2**	**Profile 3**	**Profile 4**	**Profile 5**	**Profile 6**	**Profile 7**	**Profile 8**	**Profile 9**
100% unfiltered	**Comp. A**	2.13	1.76	1.83	1.61	1.57	1.72	1.30	1.62	1.74
100% filtered	**Comp. A**	1.35	1.26	1.16	1.11	1.10	1.06	0.99	0.89	0.82
10% filtered	**Comp. A**	1.32	1.38	1.42	1.41	1.35	1.28	1.21	1.13	1.05
100% unfiltered	**Comp. B**	2.25	2.17	2.00	1.95	1.60	1.60	1.41	1.00	1.11
100% filtered	**Comp. B**	1.70	1.65	1.56	1.41	1.23	1.04	0.87	0.74	0.69
10% filtered	**Comp. B**	1.68	1.72	1.68	1.58	1.44	1.29	1.15	1.01	0.86

**Table 3.** SNR from both phantom and in vivo datasets. Phantom: 100%-sampled, without and with filtering, and 10%-sampled with filtering. In vivo: Two volunteers with the same vibration frequency and the same volunteer with two different vibration frequencies.

		**Phantom**			**In vivo**		
		**100% unfiltered**	**100% filtered**	**10% filtered**	**Volunteer 1 89 Hz**	**Volunteer 2 89 Hz**	**Volunteer 2 129 Hz**
RX1	ξ_REF MEG_+__	6.8 ± 3.6	30.7 ± 13.5	35.7 ± 14.2	31.9 ± 11.1	46.8 ± 14.6	34.9 ± 11.0
	ξ_REF MEG_−__	7.1 ± 3.6	28.7 ± 12.7	37.9 ± 15.9	34.1 ± 13.0	40.3 ± 13.1	35.1 ± 12.8
	ξ_MEG_+__	7.9 ± 3.8	26.3 ± 10.9	35.7 ± 14.1	35.7 ± 13.2	31.0 ± 9.9	36.9 ± 11.6
	ξ_MEG_−__	8.1 ± 3.9	34.5 ± 14.4	45.6 ± 19.1	28.6 ± 11.1	45.9 ± 14.6	35.6 ± 12.4
RX2	ξ_REF MEG_+__	7.2 ± 2.2	24.5 ± 7.5	38.6 ± 12.1	30.7 ± 9.3	28.4 ± 10.2	26.9 ± 8.5
	ξ_REF MEG_−__	6.7 ± 2.1	31.3 ± 9.5	33.9 ± 11.2	31.1 ± 10.2	30.8 ± 10.8	30.7 ± 10.8
	ξ_MEG_+__	6.5 ± 1.9	23.8 ± 7.3	44.1 ± 14.0	25.0 ± 7.5	22.7 ± 8.2	29.6 ± 9.8
	ξ_MEG_−__	6.3 ± 1.8	27.8 ± 8.3	57.2 ± 17.6	24.7 ± 7.9	18.0 ± 6.3	21.7 ± 7.7

### In vivo acquisitions

**Fig. 3.**
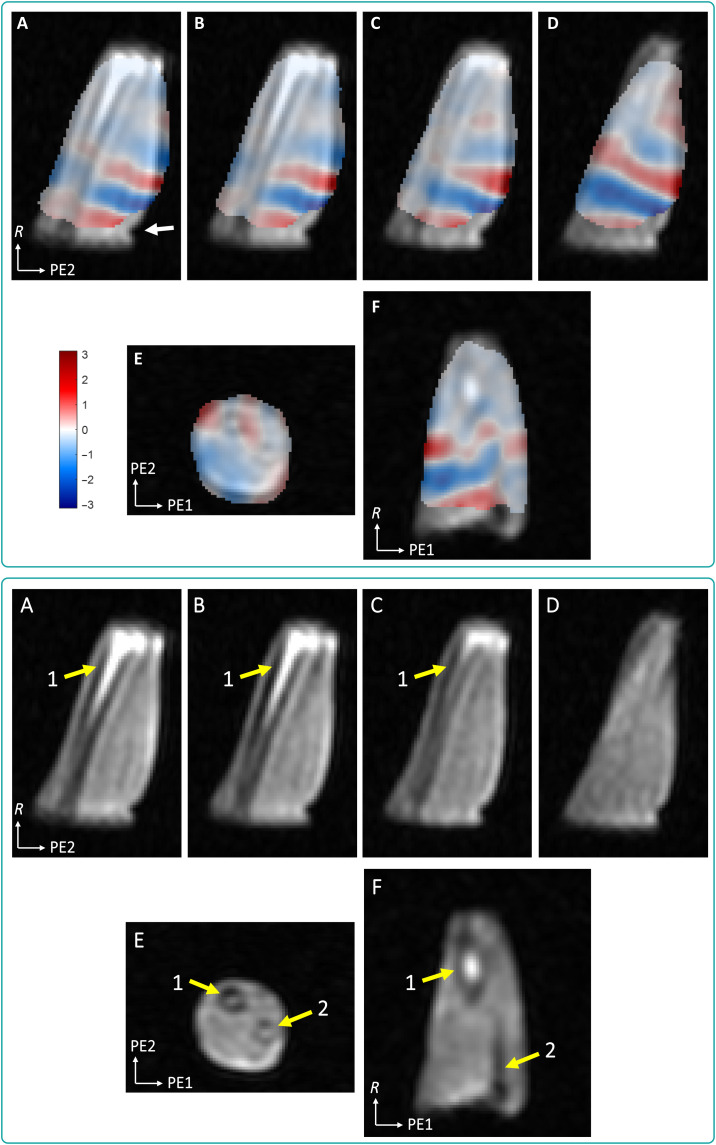
In vivo waves with an anatomic overlay. Top: Examples of in vivo wave images for an 89-Hz vibration overlayed over anatomy images [double–gradient echo steady state (DESS)], first vibration time point, of volunteer 1. Bottom: Corresponding DESS images without wave overlay. (**A** to **D**) Sagittal views at different sections of the forearm: (A to C) close to the radius and (D) section of the brachioradialis and flexor carpi muscles. (**E**) Axial view and (**F**) coronal view showing perturbations in the wavefront pattern due to the radius (yellow arrow 1) and ulna (yellow arrow 2). See also the wave movie of selected views in the Supplementary Materials. The white arrow indicates the position of the transducer cylinder. The wavelength estimated on sagittal slice B was 32.9 ± 2.1 mm, corresponding to a shear stiffness μ = 8.6 ± 1.1 kPa.

A reasonably high SNR was observed across all in vivo datasets (Table 3), comparable to phantom data in similar conditions (i.e., a 10%-filtered *k*-space). Wave propagation overlaid on anatomical images (Fig. 3) shows a good correspondence of wave pattern changes in the vicinity of the radius. The wave propagates away from the transducer location along the muscle orientation. The estimated wavelength in the region of brachioradialis and flexor carpi muscles on this dataset was 32.9 ± 2.1 mm, with a corresponding shear stiffness of 8.6 ± 1.1 kPa.

**Fig. 4.**
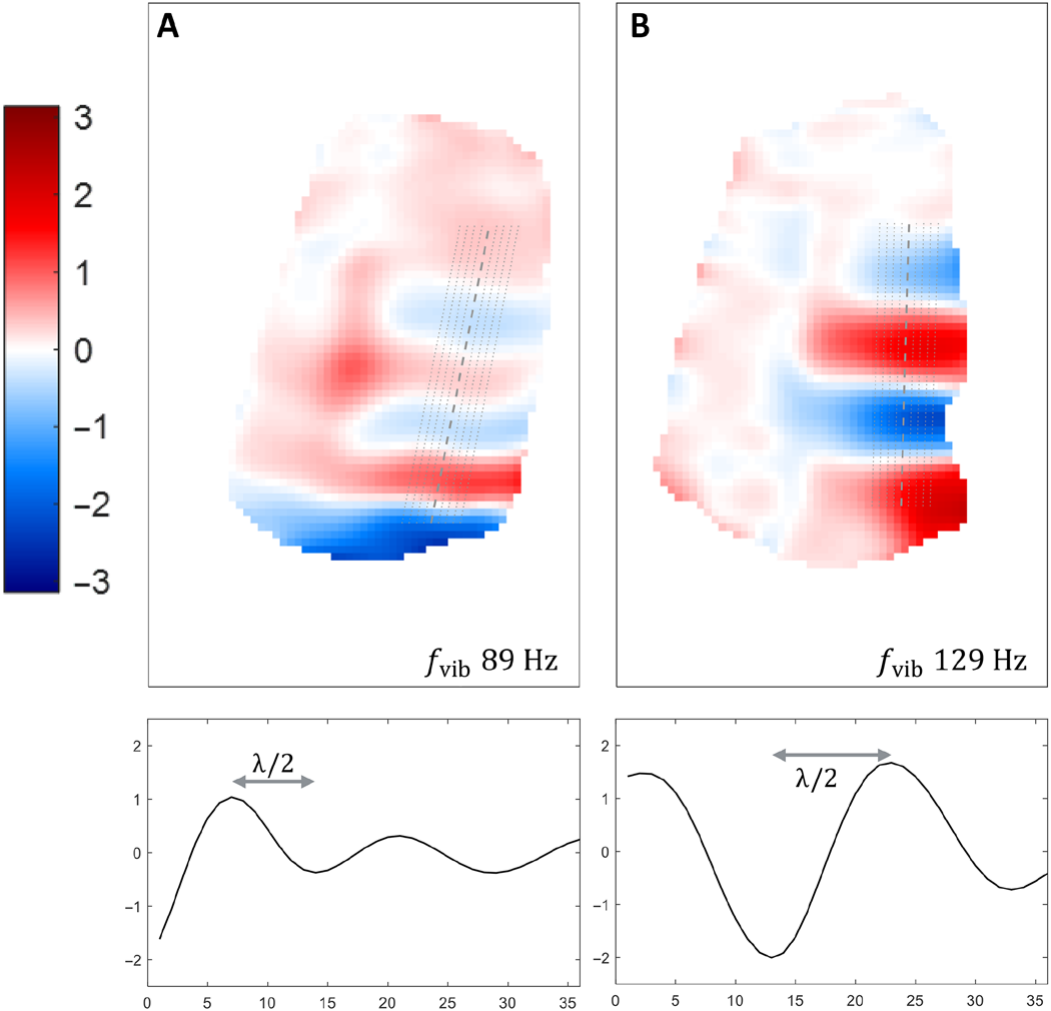
In vivo wave examples. Sagittal views with profiles used for wavelength estimation (location indicated by dotted lines), volunteer 2 (repositioned), at vibration frequencies of 89 (**A**) and 129 Hz (**B**). The plots below correspond to the dashed segments (middle profile) on the wave maps. At 89 Hz, the estimated wavelength was 28.1 ± 2.6 mm for a shear stiffness μ = 6.3 ± 1.1 kPa; at 129 Hz, the estimated wavelength was 38.9 ± 0.0 mm for a shear stiffness μ = 25.2 ± 0.0 kPa.

Figure 4 illustrates the waves at two different vibration frequencies, namely, 89 and 129 Hz, in the same volunteer (repositioned). The obtained shear stiffness is 6.3 ± 1.1 and 25.2 ± 0.0 kPa, with a wavelength of 28.1 ± 2.6 and 38.9 ± 0.0 mm, respectively.

## DISCUSSION

The results show that good motion encoding is achieved with the proposed 10%-sampling and processing method, both in the phantom and in vivo. The wave images, obtained in less than 3 min, clearly exhibit the expected propagation pattern and enable wave profile extraction for wavelength and shear stiffness estimation. The latter showed increased shear stiffness measured in phantom compartment A compared to compartment B, as expected from their composition and manual palpation. Such values are within the range observed in a previous study with similar silicone compositions (*29*). In vivo results, on the other hand, clearly show waves propagating along the muscle orientation with a pattern that follows the anatomical structures. Although few studies have investigated the forearm muscles, our results seem to provide comparable estimates of muscle shear stiffness (*30*), considering that variability exists between different muscles and volunteers (*31*, *32*). Similar to previous studies, we were able to observe dependence between the muscle properties and the vibration frequency (*33*, *34*). In addition to dispersive effects, we believe that the large increase in the shear modulus observed at 129 Hz may be due to inadvertent contraction caused by vibration. Muscle contraction conditions have been reported to greatly affect measured properties (*32*, *35*–*37*). Last, the accuracy of our results depends on the accuracy of the voxel size estimation, and wavelength estimation can be more challenging to assess when it is relatively large with respect to a given object size or a field of view (FOV), as commonly observed in most MRE reconstruction methods.

Such low-field MRE performances were made possible by hypothesizing that relevant low-frequency shear wave information is contained in the central part of *k*-space, as previously suggested (*27*, *28*). In this work, the acquired *k*-space sample distribution had a Fourier domain spacing ∆*k_i_* = 1/FOV*_i_*, *i* = *R*, PE1, PE2, equal to ∆*k_R_* = 5.5 m^−1^, ∆*k*_PE1_ = 6.5 m^−1^, ∆*k*_PE2_ = 8.8 m^−1^, respectively, for all acquisitions. In this regard, a wavelength λ = 28.1 mm, the shortest observed in our datasets, corresponds to a main spatial frequency of λ^−1^ = 35.6 m^−1^. With the hypothesis of wave propagation along *R* for illustration purposes, the main phase-encoded information of such a wave is located only ±λ^−1^/∆*k_R_* = ± 6.5 samples from the *k*-space center, which corresponds to less than 30% of the acquired *k*-space R dimension. Waves with longer wavelengths are encoded into even lower *k*-space coordinates, closer to the center. Similarly, for a given wavelength, wave information will be located within fewer samples from the center if ∆*k* increases, as is the case for our two phase-encoding directions where ∆*k*_PE1_, ∆*k*_PE2_ > ∆*k_R_*, thus allowing for greater undersampling factors. Overall, in our case, the spatial frequency of waves propagating mainly along R, with relatively small wave vector components along PE1 and PE2, is located within the chosen *k*-space sampling mask. The low-sampling pattern can therefore preserve MRE wave information, although *k*-space sampling details might need to be adapted depending on the mechanical properties under study, vibration frequency, and the targeted object geometry and imaging parameters. In comparison to previous works suggesting the idea of low-frequency *k*-space sampling (*27*, *28*), we have successfully applied it to 3D scans, achieving acceleration along two encoding directions.

In addition to scan time reduction, the proposed sampling strategy provides higher SNR and an intrinsically smoothened wave pattern, as illustrated in the 100%-sampled data (Fig. 2 and Tables 1 and 3). One can also observe that the 10%-sampling approach provides SNR and wave estimations that are similar to the filtered 100% dataset results. In all cases, the coronal orientation shows differences in wavelength between compartments A and B, with a continuous transition at the interface and possible reflections at the phantom sides. The profiles obtained on sagittal slices in the two compartments allow for a quantitative comparison between 100%- and 10%-sampled data. In general, the unfiltered 100% dataset yields noisy wave profiles (cf. Fig. 2 and Table 3) in which the wave peaks are more challenging to detect, thereby leading to a greater SD on the wavelength and, thus, on estimated shear stiffness (Table 1). This behavior is mitigated by postprocessing filtering (100%-sampled filtered dataset), allowing better wavelength characterization. In comparison to the latter case, the proposed 10%-sampling approach provides a similar error on the wavelength estimation. The average wavelength is slightly different in this case, albeit the profiles observed in Fig. 2 appear to provide more regular wave patterns with the 10% dataset. The wave amplitude extracted from the profiles of the unfiltered 100%-sampled dataset is the highest for all profiles but seem to be linked to the noise level in this dataset (Fig. 2 and Table 2). The wave amplitude is slightly lower with the proposed 10% sampling approach (for all profiles, except the first), yet it is not as reduced as in the filtered 100% dataset (Table 2). This indicates that our sampling strategy does not flatten the wave pattern, which is important for correct MRE processing. On a side note, Fig. 2 also shows rounded phantom edges (sagittal views) that occur because of the *B*_0_ inhomogeneities of the magnet at the edges of the large phantom. However, these field inhomogeneities remain far from the typical volume of interest for extremities and do not affect in vivo images (Figs. 3 and 4).

A possible limitation of this comparison is that the unfiltered 100% phantom dataset has low SNR. Although a greater number of averages should be expected to increase SNR and, hence, overall image quality, note that, with our hardware, long acquisitions become prone to various phase-affecting phenomena, such as *B*_0_ drift or shim variations due to gradient coil warming produced by imaging sequences and MEG execution. This suggests that such phenomena may also occur within the motion-sensitized scan and may not necessarily be captured in the same way by the equally long reference scan, which can compromise accuracy and consistency between scans. This feature is a key factor in correct phase retrieval and may explain some differences observed in the obtained wave patterns. For this reason, maintaining a reasonably short scan time for fully sampled acquisitions was preferred.

Note that the 3D Tukey filtering (along R, PE1, and PE2) of 10% data provides very similar results to 10% data with only 1D Tukey filtering along R (cf. Fig. 2C and fig. S1) since *k*-space data are already restricted to the central region along PE1 and PE2. These results suggest that it should also be possible to sample a more restricted central *k*-space portion in the readout direction (i.e., lower resolution). For instance, in the example above, to acquire only the central 30% of the *k*-space R direction, the receiver acquisition window *T*_acq_ would be shortened by 70%. Consequently, TE would be reduced by 35% of *T*_acq_, thus reducing T2* dephasing effects and gaining SNR, which can then be traded for scan time reduction (e.g., less averaging). While, in our case, such a gain would have a limited effect because of an already short *T*_acq_ of 2.3 ms, cases with longer acquisition windows, due to more readout points or lower readout bandwidth, could benefit from this approach.

Our innovative method enables, at low field, good encoding of shear waves in vivo in humans. In this work, one motion-encoded direction is obtained at five vibration time points in less than 3 min for a 3D dataset. The low *k*-space sampling approach helps exploit the SNR benefit of a simple Cartesian 3D gradient echo sequence. This, in turn, represents a promising path for future work since standard high-field MRE acceleration 2D approaches cannot be easily transposed to low-field applications because of the intrinsic lower SNR and shorter T1s available in this regime. Furthermore, a 3D acquisition allows 3D data processing and displays of any orthogonal view if the acquired voxels are isotropic or almost isotropic. 3D *k*-space can be accurately interpolated in all three directions by a variable factor (up to 2), corresponding to variable reconstructed voxel sizes [if the SNR is sufficient (*23*)], which could be useful for obtaining optimal conditions for wavelength/voxel size in 3D reconstruction techniques (*38*–*42*).

In this work, we chose to encode only one motion component, assuming that it is a reasonable approach when considering the anisotropy of skeletal muscles. Even by encoding the other two spatial directions, total scan time would still be short for such a low magnetic field. These additional encoding orientations might be required in the future, depending on the organs/pathology of interest.

Last, a reference dataset was needed in our experiments, which increased scan time. With multiple, successive vibration time points, one can use the same reference if the scan time is sufficiently short to prevent *B*_0_ variations over time, thereby making the experiment more time-efficient. To obtain *N* time points, a total scan time of (*N* + 1)*T*_ξ±_ is hence required. That said, the proposed motion-encoding approach, in principle, should not require an additional reference scan, thanks to subtracting the MEG^−^ from the MEG^+^ sensitized dataset. However, we observed an undesired phase gradient that clearly appears on the reference scan, which we suspect originated from an imperfectly symmetrical execution of the two MEG polarities. We note that our processing approach (Fig. 1) was nevertheless successful in removing the spurious phase effects that otherwise would have largely covered the encoded wave information. The choice of using complex data is particularly appropriate for handling multiple phase wraps in the intermediate steps, without introducing inconsistencies. Further work will be pursued to investigate and mitigate the origin of the observed spurious phase gradient, which may allow for skipping this extra step at the benefit of total scan time.

Overall, in our conditions, it is reasonable to favor acquisition acceleration by a factor of 10 and intrinsically discard high-frequency noise to obtain high SNR, which is key for reliable MRE (*29*), rather than to apply postprocessing filters to fully sampled scans. Filtering is quite common in MRE reconstruction pipelines, especially in those relying on spatial derivatives that are rather sensitive to noise (*43*). Following this feasibility study, future work should explore the performance limits of 3D *k*-space undersampling and the possibilities of generalizing 3D MRE at high fields.

Unprecedented at low field, our work enables motion encoding for MRE in vivo in humans within just a few minutes. This was achieved by sampling only 10% of 3D *k*-spaces via a simple gradient echo Cartesian acquisition, applying an appropriate spurious-phase compensation strategy and processing, as well as using an optimized RF coil. Our results in a phantom provided similar, or even better, wave information than a fully sampled acquisition. The waves obtained in the forearm muscles exhibited a clear propagation pattern consistent with the anatomy and expected mechanical behavior. Building upon this foundational work, future studies will aim to perform MRE at low field in other organs and further improve the acquisition strategy via more advanced sequences.

As for future implementations, different avenues are foreseen. On the one hand, the demonstration of our method in the forearm highlights the potential of dedicated low-field MRI devices in MSK applications for human limbs. Such technology represents an opportunity for small-footprint scanners with reduced costs and requirements, thus enabling more accessible and efficient clinical workflows with devices located directly in the physician’s office or in resource-poor areas. This opens up perspectives in diffuse myopathies, healing monitoring after surgery, or weight-bearing applications. On the other hand, our method should not be considered as being restricted to MR scanners designed for extremities, as the low magnetic field intensity is not strictly dependent on the size of the MRI magnet. A whole-body low-field MR scanner can benefit from an adapted version of our method to perform MRE in CLD patients with iron overload. Typically, such a condition often leads to unsuccessful or unreliable diagnostic outcomes for liver MRE in common high-field scanners because of magnetic susceptibility issues (*5*–*7*, *9*). The recent renewed industrial interest in middle- and low-field MR scanners is a promising ground for making such MRE applications more accessible for clinical use.

## MATERIALS AND METHODS

### Phantom

The MRE phantom was created using a bicomponent silicone rubber (Eurosil 4 A+B, Schouten SynTec, the Netherlands) and a 3D-printed mold, following the method described in (*29*). The phantom (~10 cm by 11 cm by 14 cm) consisted of two 5-cm-wide compartments with different mechanical properties, obtained by varying the amount of softener (S) in the silicone mixture (1A:1B:2.5S and 1A:1B:1.5S). As described previously, this material can reproduce the range of shear stiffness typically observed in biological tissues, with rather strong frequency-dependent viscoelastic and attenuation behavior.

### Hardware

**Fig. 5.**
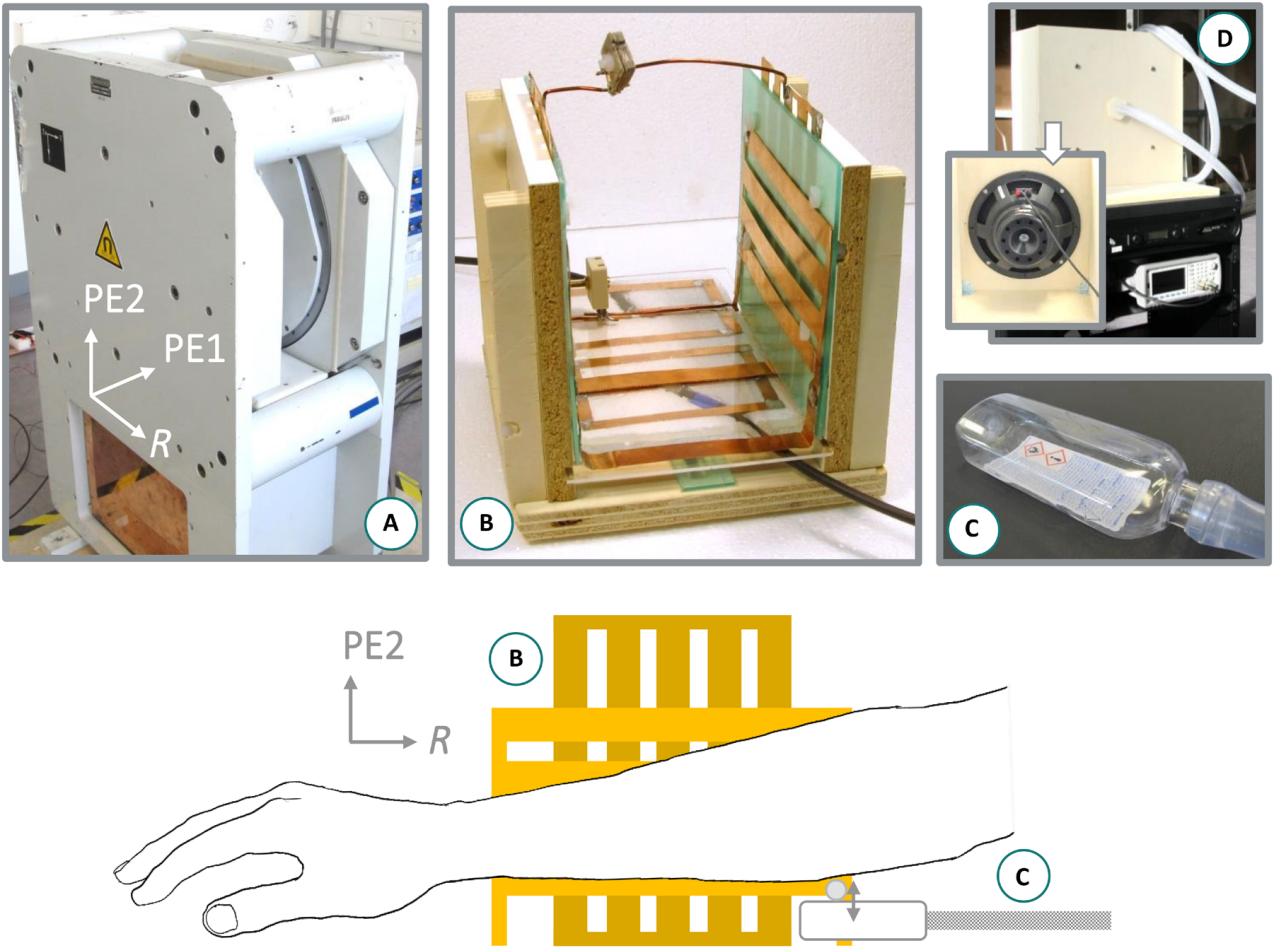
Experimental setup. Top: Experimental equipment: (**A**) 0.1-T biplanar magnet, (**B**) open biplanar quadrature RF coil (RX1: strips parallel to R; RX2: strips parallel to PE2), (**C**) pneumatic transducer, and (**D**) vibration system. Bottom: Schematic of arm and transducer positioning in the coil, sagittal view; R, PE1, and PE2 indicate the 3D scan readout, PE1, and PE2 directions, respectively.

All MR scans were performed on a resistive, biplanar 0.1-T system (EAR54L, Drusch & Cie, France) designed for extremities imaging (*44*, *45*). By design, the scanner has three open sides and a horizontal *B*_0_ produced by two 60-cm-diameter magnet coils, with an 18-cm interplane gap (Fig. 5A). It does not use magnetic or electromagnetic (Faraday cage) shielding, yet efficient noise reduction can be achieved by connecting the grounds of the magnet planes with aluminum foil, acting as a partial shield. An optimized quadrature biplanar RF volume coil was used (Fig. 5B), as described in (*46*). Both coil channels (RX1 and RX2) were tuned to 4.333 MHz and exhibited good geometrical decoupling of −38.0 dB (coil RX1/RX2: *S*_11_, −4.5/−4.2 dB; bandwidth_-3dB_, 15.8/13.0 kHz; Q 274/333). Coil RX1 was interfaced in transceive mode using a TR switch (NMR Service, Germany) and an RF power amplifier (BT0500-AlphaS, Tomco Technologies, Australia). Both channels were connected to two custom high-impedance preamplifiers (*47*) for signal reception. All MRI sequences were programmed on a Cameleon 3 spectrometer (RS^2^D, France).

The used custom vibration system, based on a loudspeaker and an air waveguide (Fig. 5D), is described in detail in (*29*) and is similar to the examples described in (*13*, *48*). In the present case, the lowest useful waveguide resonant frequencies were 49, 89, and 129 Hz. A soft polyethylene terephthalate flask was positioned at the end of the waveguide and acted as a transducer to convey motion to the phantom or human body parts (Fig. 5C). For in vivo imaging, a plastic cylinder (Ø 0.5 cm) was further attached to the transducer surface to reduce the vibration source surface and avoid bulk motion of the elbow. The transducer was positioned at the edge of the coil to ensure wave propagation toward the magnet’s isocenter.

### Low central *k*-space sampling for low–spatial frequency shear wave acquisition

**Fig. 6.**
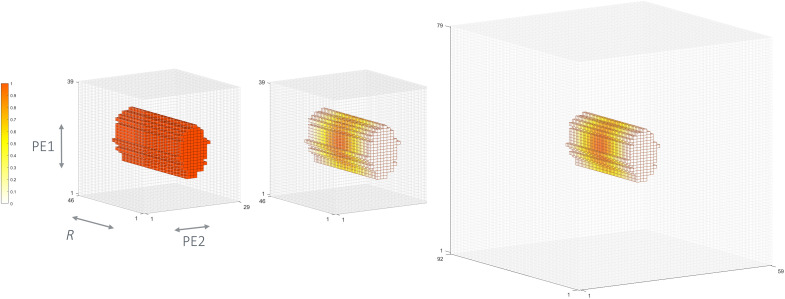
Acquired *k*-space and preprocessing steps. Left: 3D view of the acquired 10%-sampled *k*-space. Middle: *k*-space voxel contribution after applying the 3D Tukey filter. Right: 3D zero-filling of the filtered *k*-space. R, readout or frequency encoding direction; PE1, PE1 direction; PE2, PE2 direction.

Cartesian *k*-space sampling was performed using a random Gaussian pattern (*13*, *22*), with only 10% of central phase-encoding steps acquired (along PE1 and PE2 in Fig. 6). This drastic undersampling scheme, although not appropriate for conventional anatomical imaging, has the obvious advantage of accelerating acquisition time by focusing mostly on low spatial frequencies, which are particularly relevant in capturing shear wave displacement. The Fourier domain spacing along direction *i* is ∆*k_i_* = 1/FOV*_i_*, where FOV is the imaging field of view. For a given ideal sinusoidal wave with spatial wavelength λ, the main Fourier component along *i* would be encoded in the complex MR signal at coordinates ±λ^−1^. Hence, whenever *k*-space frequencies are sampled up to the coordinate ∣*K_i_* * ∆*k_i_*∣ > λ^−1^, with *K_i_* being a sufficiently large sampling rate along *i*, the wave should be correctly retrieved. Since MRE shear waves should exhibit a sufficiently long wavelength with respect to voxel size, information of such waves will be located in low *k*-space frequencies. Of course, the way shear waves are captured will depend on the mechanical properties under study and the chosen FOV parameters. The highest *k*-space frequencies are not expected to contain any useful MRE information. On the one hand, this would imply that the shear wavelength is close to the voxel size, which would not be exploitable and is usually avoided in MRE. Therefore, an appropriate sampling scheme tailored to MRE wave information can enable great scan acceleration by focusing on the appropriate low-frequency *k*-space coordinates only, without the risk of discarding meaningful information and with the added benefit of acquiring less noise.

Despite all of these advantages in MRI, they come with some limitations, and heavy undersampling can produce phase artifacts in the image domain. In our pipeline, however, since we subtract two datasets with inverted MEGs and consider only the resulting motion-encoded phase images, the low-sampling artifacts are successfully canceled out. To evaluate the accuracy of this approach, a fully sampled dataset was also acquired in the phantom (reference + first vibration time point). The proposed 10% sampling scheme was ultimately used to scan the forearm of healthy human volunteers with 89- and 129-Hz vibration frequencies.

### MRE experiments

Wave propagation was encoded via a custom, Cartesian, gradient echo–based sequence using trapezoidal bipolar MEGs. To reduce TE, fractional encoding was used (*49*), with a MEG frequency *f*_MEG_ higher than the lowest chosen vibration frequencies *f*_vib_ of 49 and 89 Hz only (see Table 4). Sequence TR was set to a multiple of 1/*f*_vib_, and continuous vibration was synchronized to the sequence via a TTL trigger at the beginning of each TR. Datasets were acquired over one to five time points covering the period of the vibration by varying the phase of the sinusoidal signal (0°, 72°, 144°, 216°, and 288°) fed to the loudspeaker. A dataset without mechanical vibration was also acquired as a reference. The MR sequence acquired each time point sequentially with a default (MEG^+^) and an inverted MEG polarity (MEG^−^) using MEG amplitude of 8.15 mT/m. By subtracting the two datasets, motion-encoding efficiency doubles, while phase effects due to *B*_0_ inhomogeneities, eddy currents, or imaging gradient imperfections automatically cancel out (*50*–*52*). For all scans, one motion-encoding direction was chosen with an orientation parallel to the transducer vibration direction, i.e., along the second phase-encoding direction (Fig. 5). For each vibration time point and for the reference, a total of four 3D *k*-spaces were acquired in a single acquisition: two encoding polarities for two receive channels.

**Table 4.** MRE sequence parameters for phantom scans (with 49-Hz vibration) and in vivo scans of a human arm (with 89- or 129-Hz vibration). R/PE1/PE2, readout/PE1/PE2 directions.

	**Phantom**	**Arm**	**Arm**
Vibration frequency	49 Hz	89 Hz	129 Hz
MEG frequency	89 Hz	106 Hz	129 Hz
MEG amplitude	8.15 mT/m	8.15 mT/m	8.15 mT/m
MEG ramp time	400 μs	400 μs	400 μs
MEG plateau time	4.818 ms	3.917 ms	3.076 ms
Flip angle	30°	30°	30°
TE/TR	14.33/20.41 ms	12.52/22.47 ms	10.84/15.50 ms
Readout bandwidth	20 kHz	20 kHz	20 kHz
Field of view (R × PE1 × PE2)	180.5 × 153.0 × 114.0 mm^3^	180.5 × 153.0 × 114.0 mm^3^	180.5 × 153.0 × 114.0 mm^3^
Acquisition matrix (R × PE1 × PE2)	46 × 39 × 29	46 × 39 × 29	46 × 39 × 29
Acquired voxel size	3.9 × 3.9 × 3.9 mm^3^	3.9 × 3.9 × 3.9 mm^3^	3.9 × 3.9 × 3.9 mm^3^
Reconstructed matrix	92 × 79 × 59	92 × 79 × 59	92 × 79 × 59
Reconstructed voxel size	1.9 × 1.9 × 1.9 mm^3^	1.9 × 1.9 × 1.9 mm^3^	1.9 × 1.9 × 1.9 mm^3^
Number of averages	10	6	8
Scan time per vibration time point *T*_ξ±_	46 s (10% sampling) 7 min 41 s (100%)	30 s (10%)	28 s (10%)

The acquisition parameters of the MRE scans are summarized in Table 4. For one encoding direction, scan time per vibration time point, *T*_ξ±_, yielding two opposite-encoded datasets in vivo for two receiver channels, was 30 and 28 s for 89- and 129-Hz vibration, respectively. As detailed below, a reference time point is required for data processing. Thus, the duration of a complete protocol acquiring *N* time points with our method was (*N* + 1)*T*_ξ±_, i.e., 3 min in vivo for five time points. In addition, a 3D double–gradient echo steady-state sequence was used to obtain images of the phantom and arm (flip angle: 30°; TE1, TE2/TR: phantom, 10.0, 22.0/27.8 ms and arm, 3.3, 26.5/30.0 ms, 50% undersampling; scan time: phantom, 2 min and 6 s and arm, 1 min and 25 s). For this sequence, the combination of multichannel data into the magnitude image was done as described in (*46*). The FOV and matrix size were the same for all scans. In vivo experiments were conducted after informed consent was obtained and in accordance with the Declaration of Helsinki (ethics approval EKNZ/2022-00348).

### Data processing

All processing was done using custom MATLAB scripts (MathWorks, USA). For each vibration time point, the data for each separate receive channel (RX1 and RX2) were processed as follows. The recorded (raw) 3D *k*-spaces were first filtered with a 3D Tukey window (*r* = 1) before padding the resulting *k*-spaces with zeros to increase their size and improve image resolution by a factor of two in all three dimensions (Fig. 6) (*23*). After Fourier transform, the complex 3D images, ξ*_i_*, were processed as described in Fig. 1, their magnitude being defined as ∣ξ*_i_*∣ and phase φ*_i_* = arg (ξ*_i_*). The total, motion-encoded phase φ_MEG_ was retrieved by subtracting the opposite motion-encoding polarities (MEG^+^, MEG^−^) obtained by computing the phase of the complex-conjugate multiplication of ξ_MEG_+__ and ξ_MEG_−__ (Fig. 1, step 1)

ξMEG=ξMEG+∙ξMEG−¯ (1)

$$\varphi_{\text{MEG}}=\text{arg}(\xi_{\text{MEG}})$$ (2)

The reconstructed phase of the reference complex data ξ_REF_ (i.e., without vibration), also acquired with two encoding polarities, was obtained with the same workflow to provide φ_REF_

$$\xi_{\text{REF}}=\xi_{\text{REF}\;{\text{MEG}}_{+}}∙\bar{\xi_{\text{REF}\;{\text{MEG}}_{-}}}$$ (3)

$$\varphi_{\text{REF}}=\text{arg}(\xi_{\text{REF}})$$ (4)

To correct for spurious phase gradients (cf. Fig. 1, particularly noticeable on the reference phase image φ_REF_, which in the absence of such effects is expected to be flat), φ_REF_ was then subtracted from φ_MEG_ to generate a corrected, motion-encoded phase φ_RX_ (Fig. 1, step 2)

$$\varphi_{\text{RX}}=\text{arg}(\xi_{\text{MEG}}∙\bar{\xi_{\text{REF}}})$$ (5)

As receive channels RX1 and RX2 exhibit different spatial sensitivities, a weighted magnitude *w*_RXi_ (*i* = 1,2) was defined before combining the motion-encoded images, relying on the ratio of the normalized, reference images $\xi_{\hat{\text{REF}\;\text{RXi}}}$ as follows

$$w_{\text{RXi}}=\frac{|\;\xi_{\hat{\text{REF}\;\text{RXi}}}|}{|\;\xi_{\hat{\text{REF}\;\text{RX}1}}|+|\;\xi_{\hat{\text{REF}\;\text{RX}2}}|}$$ (6)

Two weighted, motion-encoded images with corrected phases can hence be obtained for each channel

$$\xi_{\text{RXi}}=w_{\text{RXi}}\;e^{i\;\varphi_{\text{RXi}}}$$ (7)

Ultimately, the complex images of the two channels are summed, and the final wave information Ψ can be retrieved as the phase of the latter as follows (Fig. 1, step 3)

$$\Psi =\text{arg}(\xi_{\text{RX}1}+\xi_{\text{RX}2})$$ (8)

In vivo phase images were unwrapped in 3D using the MATLAB function robustunwrap (*53*). The resulting phase images were masked on the basis of an empirical threshold of $|\xi_{\text{REF}\;{\text{MEG}}_{+}}∙\bar{\xi_{\text{REF}\;{\text{MEG}}_{-}}}|$, excluding voxels outside the object. Mean SNR and the respective SDs were calculated over the entire phantom and arm (*29*), for each receive channel and dataset.

Wave profiles were extracted from nine parallel segments positioned manually on sagittal phase images (step 3) and along the direction of wave propagation (*32*, *34*, *36*, *37*, *54*–*56*). Bilinear interpolation was applied to these profiles before further analysis (Fig. 2). Half the wavelength, λ/2, was estimated on each wave profile as the distance between a positive and a negative peak, and the average wavelength over the nine segments was calculated together with its SD. In addition, wave amplitude, i.e., intensity difference between the positive and negative peaks, was computed. For each profile, shear stiffness μ was calculated from wavelength λ using the assumptions of linear elasticity, incompressibility, isotropy, and homogeneity, defined in Eq. 9 (*57*)

$$\mu =\rho ∙\lambda^{2}∙f_{\text{vib}}^{2}$$ (9)

where ρ is muscle and silicone density, assumed as 1000 kg/m^3^ (*32*, *37*, *55*–*58*). Stiffness was given as mean ± SD over the nine wave profiles.

## Notes

View/request a protocol for this paper from *Bio-protocol*.
