# Supplementary material for: Fast acquisition of propagating waves in humans with low-field MRI: Toward accessible MR elastography
Source: Sci Adv. 2022 Sep 9;8(36):eabo5739. doi: 10.1126/sciadv.abo5739 (PMC9462689; doi:10.1126/sciadv.abo5739)
Supplement: Supplementary file 1 — Fig. S1 [file sciadv.abo5739_sm.pdf]

Science Advances
AAAS

Supplementary Materials for

Fast acquisition of propagating waves in humans with low-field MRI: Toward accessible MR elastography

Maksym Yushchenko *et al.*

Corresponding author: Maksym Yushchenko, maksym.yushchenko@unibas.ch

Sci. Adv. **8**, eabo5739 (2022)
DOI: 10.1126/sciadv.abo5739

The PDF file includes:

Fig. S1
Legend for movie S1

Other Supplementary Material for this manuscript includes the following:

Movie S1

Fig. S1.

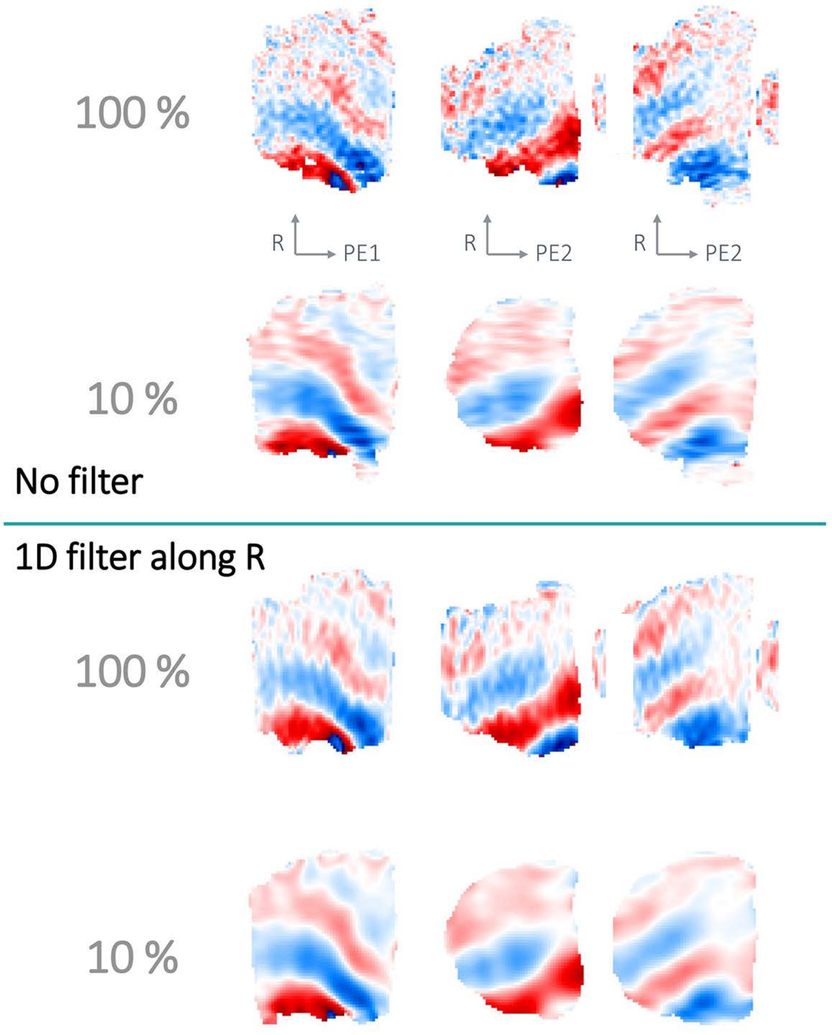

Filter effect comparison on phantom data. Comparison of slices in Fig. 2 obtained in the phantom without k-space filtering (above) and with a 1D filter along the readout (R) direction (below), for 100% and 10% sampled data.

Movie S1.

In vivo waves propagating in the arm for an 89-Hz vibration. The movie shows the wave data of five acquired timepoints overlayed over anatomy images of volunteer 1 (cf. Fig. 3), which presents the first vibration timepoint), on a sagittal, a coronal and an axial view.
